# Trends in diabetes-related sickness absence and social insurance costs in Poland: a national registry-based study, 2020–2024

**DOI:** 10.3389/fpubh.2026.1903345

**Published:** 2026-10-13

**Authors:** Piotr Artur Winciunas, Kuba Sękowski, Agnieszka Mazurek, Wojciech S. Zgliczyński, Mateusz Jankowski, Justyna Grudziąż-Sękowska

**Affiliations:** School of Public Health, Center of Postgraduate Medical Education, Warsaw, Poland

**Keywords:** diabetes mellitus, health burden, Poland, productivity loss, registry data, sickness absence, social insurance expenditures

## Abstract

**Introduction:**

Diabetes mellitus is recognized as a major contributor to productivity losses, yet nationwide evidence on diabetes-related sickness absence in Central and Eastern Europe has been limited. This study was designed to characterize trends in sickness absence and rehabilitation benefits due to type 1 and type 2 diabetes in Poland between 2020 and 2024 using national social insurance registry data.

**Methods:**

All sickness absence and rehabilitation benefit certificates issued in 2020–2024 for adults covered by the Polish Social Insurance Institution (ZUS) were analyzed, with diabetes identified by ICD-10 codes E10 (type 1) and E11 (type 2). Annual numbers of certificates, total days of absence, crude rates per 100,000 population, and social insurance expenditures in PLN and EUR were calculated and stratified by sex, age group and region.

**Results:**

Among 16.6 million insured adults, days of sickness absence due to type 1 diabetes were reduced from 300,387 in 2020 to 232,976 in 2024 (−22.4%), while those due to type 2 diabetes were reduced from 520,090 to 488,531 (−6.1%). Between 2020 and 2024, the reduction in type 2 diabetes—related absence was driven mainly by women (−20.2%), whereas only a marginal decline was observed among men (−1.5%). Despite these declines in days of absence, yearly social insurance expenditures were increased by 16.1% for type 1 diabetes and by 39.8% for type 2 diabetes, reaching approximately 6.44 and 15.03 million EUR in 2024, respectively, with up to four-fifths of total costs being attributed to men. Marked regional variation was observed, with voivodeship-level rates differing by more than twofold and rising diabetes-related absence being recorded in some regions despite national declines.

**Conclusions:**

In Poland, diabetes-related sickness absence declined between 2020 and 2024, while social insurance expenditures increased substantially, particularly for type 2 diabetes and among men. These findings highlight the need for targeted workplace and clinical interventions.

## 1 Introduction

Diabetes mellitus is one of the most prevalent chronic diseases worldwide and a leading cause of morbidity, disability, and premature mortality (1, 2). The global burden of diabetes has increased substantially over recent decades (2, 3). According to the Global Burden of Disease Study, global prevalence rose from 476 million in 2017 to a projected 571 million by 2025, accompanied by rising incidence, mortality, and disability-adjusted life-years (DALYs) across virtually all regions (4, 5). The International Diabetes Federation estimated that 589 million adults aged 20–79 years were living with diabetes globally in 2024, a figure projected to reach 853 million by 2050 (1). Diabetes caused 3.4 million deaths in 2024 and was responsible for an estimated USD 1.015 trillion in global health expenditure, representing a 338% increase over the past 17 years (6). The rising prevalence of diabetes has been described as the fastest-growing global public health concern, with projections indicating a further surge to 643 million affected individuals by 2030 and 783 million by 2045 (2).

Poland is a European Union country with a high and growing burden of diabetes (7, 8). According to a combined analysis of national databases, the estimated prevalence of diabetes (diagnosed and undiagnosed cases) in Poland was 6.97% in 2013, corresponding to approximately 2.68 million people. By 2014–2017, the number of people with diagnosed diabetes rose from 2.11 million to 2.53 million (6.58% of the population), with women constituting the majority of cases. Current estimates indicate that over 3 million Poles are affected by diabetes, representing approximately 8% of the population (7, 8). A continuous growth of both the incidence and the prevalence of diabetes has been predicted, with projections suggesting that the number of people with diabetes in Poland may reach 4.2 million by 2030 (4).Diabetes belongs to the national health priorities defined in the regulation of the Minister of Health, and if poorly controlled, it leads to the development of many serious complications, both acute and chronic, that exclude people of productive age from effective professional activity.

Diabetes imposes a substantial economic burden, comprising both high direct medical costs and significant indirect losses from reduced productivity, sickness absence, disability, and premature mortality. Studies from the U.S. (9) and several European countries (10–13) consistently show that indirect costs, particularly those linked to work productivity, represent a major share of the total financial impact of diabetes.

Sickness absence (absenteeism) is one of the most measurable dimensions of diabetes-related productivity loss (14). Evidence from multiple countries has demonstrated that individuals with diabetes experience significantly higher rates of work absence than non-diabetic populations. A large Swedish registry-based study comprising 413,000 people with type 2 diabetes and 4.9 million person-years of follow-up demonstrated that 47% of individuals with type 2 diabetes had any sickness absence, compared with 26% of matched controls, and that diabetes-related complications were a key driver of absence days—with stroke adding 102 days, end-stage renal disease 70 days, and severe vision loss 56 days per year (15). A Danish retrospective cohort study with up to 17 years of follow-up showed that people with both type 1 and type 2 diabetes had significantly higher hazard ratios (HR) for sickness absence (type 2 diabetes: women HR 1.46; men HR 1.64), a higher risk of disability pension (type 2 diabetes: women HR 1.78; men HR 2.11), and, among women with type 2 diabetes, a lower rate of return to work (16). An Israeli study confirmed that diabetic employees had significantly more days of absence than non-diabetic controls (8.5 vs. 2.7 days per year), with diabetes complications associated with a threefold increase in absence days (15.7 vs. 5.7 days) (17). A systematic review on presenteeism attributed to diabetes found that hypoglycemia, diabetic neuropathy, and mood disorders were the main factors inducing reduced productivity at work (18). A recent integrative review confirmed that diabetes mellitus is associated with substantial productivity losses, increased absenteeism, presenteeism, and early retirement, and that occupational factors such as shift work, long working hours, and chronic stress act as additional determinants of the disease (19).

In Poland, sickness absence is compensated within the social insurance system (20). Under social insurance, working individuals can receive benefits for short-term sickness (sick leave benefit) or long-term incapacity for work (rehabilitation benefit), depending on the duration of incapacity. A statutory retirement age in Poland is 60 years for women and 65 years for men (20).

Since December 1, 2018, doctors in Poland issue sick leave certificates only in electronic form (ZUS ZLA), which are recorded in a dedicated IT system managed by the Social Insurance Institution (Zakład Ubezpieczeń Społecznych, ZUS) (20). The social insurance registry provides standardized, nationwide data on causes of sickness absence (coded according to the International Classification of Diseases, ICD-10), duration (absence days), the number of certificates issued, and the insured person's sex and age (20). Population-based data on sickness absence due to diabetes may provide essential information for clinicians and policymakers on the impact of diabetes on work productivity and support health system planning and the design of targeted prevention and return-to-work strategies (15–19). However, despite the high and growing burden of diabetes in Poland, there is a lack of population-based studies assessing trends in sickness absence due to diabetes in this country.

Therefore, this study aimed to quantify the burden and examine temporal and demographic trends in diabetes-related sickness absence in Poland using nationwide Social Insurance Institution registry data.

## 2 Material and methods

### 2.1 Data sources and measurement

The data used in this analysis were obtained from the official statistical datasets managed by the Department of Statistics and Actuarial Forecasts of the Social Insurance Institution (Zakład Ubezpieczeń Społecznych, ZUS), through a public information request for scientific purposes (21). The data were routinely collected and maintained by ZUS as part of the administration of the national social insurance system; no data were collected directly from study participants by the authors. The 2020–2024 study period was selected to provide a 5-year analysis based on consistent and comparable nationwide registry data. The digital dataset included all cases of sickness absence among 16.6 million adults covered by Poland's social insurance system. The study population comprised persons covered by the Polish social insurance system and eligible for sickness absence benefits.

In accordance with the Polish social insurance system, the study focused on short-term benefits from the sickness fund, specifically sick leave benefits and rehabilitation benefits, with eligibility determined via medical certification by authorized physicians. Sick leave certificates are issued by general practitioners or specialists, whereas rehabilitation benefit certificates are provided by ZUS medical examiners. Causes of incapacity were classified according to the primary diagnosis recorded on the medical certificate using the International Classification of Diseases, 10th Revision (ICD-10) (22). The analysis included certificates with type 1 diabetes (E10) or type 2 diabetes (E11) recorded as the primary diagnosis causing work incapacity (22). No additional individual-level inclusion or exclusion criteria were applied. Given the distinct demographic and clinical characteristics of type 1 and type 2 diabetes, all analyses were conducted and reported separately for the two diabetes types.

Sick leave variables included the annual number of medical certificates, total sick leave days, average duration of absence, and total social insurance expenditures (in thousands of PLN). The number of sickness certificates refers to the number of medical certificates issued and does not necessarily correspond to the number of independent sickness absence episodes - consecutive or renewed certificates could not be identified separately in the available aggregate data. Rehabilitation benefit data comprised national and regional (voivodeship) numbers of medical certificates. PLN values were converted to euros (EUR) using National Bank of Poland average annual exchange rates (2020: 1 EUR = 4.44 PLN; 2024: 1 EUR = 4.30 PLN) (23).

### 2.2 Ethics

The study protocol was approved by the Ethics Committee at the Center of Postgraduate Medical Education, Warsaw, Poland (approval no. 89/2025, October 8, 2025). The requirement for informed consent did not apply because the analysis was based on anonymized, aggregated data obtained from publicly accessible registries. No personal identifiers were used, and no individuals were approached directly. The study was conducted in accordance with the Declaration of Helsinki.

### 2.3 Statistical analysis

Descriptive statistics were used. Data analysis was conducted using IBM SPSS version 29. Data were analyzed separately for type 1 and type 2 diabetes. Results were stratified by sex, age group, and administrative region/subregion (voivodeship or county). The study presents both absolute counts (number of certificates and total days of absence) and crude rates per 100,000 population, calculated using the corresponding population denominators. At the county level, crude rates per 10,000 population were presented. Demographic population data were derived from the Demographic Yearbooks of the Central Statistical Office (24).

## 3 Results

The number of sick leave days due to type 1 diabetes decreased from 300,387 in 2020 to 232,976 in 2024 (a reduction of 22.4%) (Table 1). Between 2020 and 2024, the number of sick leave days related to type 1 diabetes declined by 19.1% among males and by 30.0% among females.

**Table 1:** Changes in the number of sick leave days and sick leave certificates due to diabetes type 1 (ICD-10: E10) or type 2 (ICD-10: E11) among adults in Poland between 2020 and 2024.

	Number of sick leave days due to diabetes							Number of sick leave certificates due to diabetes						
	2020	2021	2022	2023	2024	% change 2024–2020	% change 2024–2023	2020	2021	2022	2023	2024	% change 2024–2020	% change 2024–2023
Type 1 diabetes														
**Overall**	300,387	243,720	229,767	232,821	232,976	−22.4	0.1	20,111	17,088	16,845	17,855	18,501	−8.0	3.6
Males	207,477	178,744	166,012	169,983	167,923	−19.1	−1.2	13,414	11,928	11,477	12,188	12,324	−8.1	1.1
Females	92,910	64,976	63,754	62,838	65,043	−30.0	3.5	6,697	5,160	5,367	5,667	6,176	−7.8	9.0
Type 2 diabetes														
**Overall**	520,090	505,479	475,697	491,221	488,531	−6.1	−0.5	37,564	37,790	37,123	38,889	39,285	4.6	1.0
Males	392,778	393,141	377,954	390,811	386,880	−1.5	−1.0	27,850	28,752	28,507	29,552	29,772	6.9	0.7
Females	127,305	112,338	97,743	100,393	101,630	−20.2	1.2	9,713	9,038	8,616	9,336	9,511	−2.1	1.9

The number of sick leave days due to type 2 diabetes decreased from 520,090 in 2020 to 488,531 in 2024 (a reduction of 6.1%). Between 2020 and 2024, this number declined by 1.5% among men and by 20.2% among women.

The number of sick leave certificates issued for type 1 diabetes decreased from 20,111 in 2020 to 18,501 in 2024 (a 8.0% decrease). In contrast, for type 2 diabetes, an increase was observed—from 37,564 to 39,285 (a 4.6% rise). Between 2020 and 2024, the number of certificates related to type 1 diabetes decreased by 8.1% among men and by 7.9% among women. For type 2 diabetes, the number of certificates among women decreased by 2.1%, whereas among men it increased by 6.9% (Table 1).

The monthly number of sick leave certificates due to diabetes in 2024 is shown in Figure 1. The lowest monthly numbers were recorded in August for type 1 diabetes and in December for type 2 diabetes, while the highest numbers for both types were observed in January (Figure 1).

**Figure 1:**
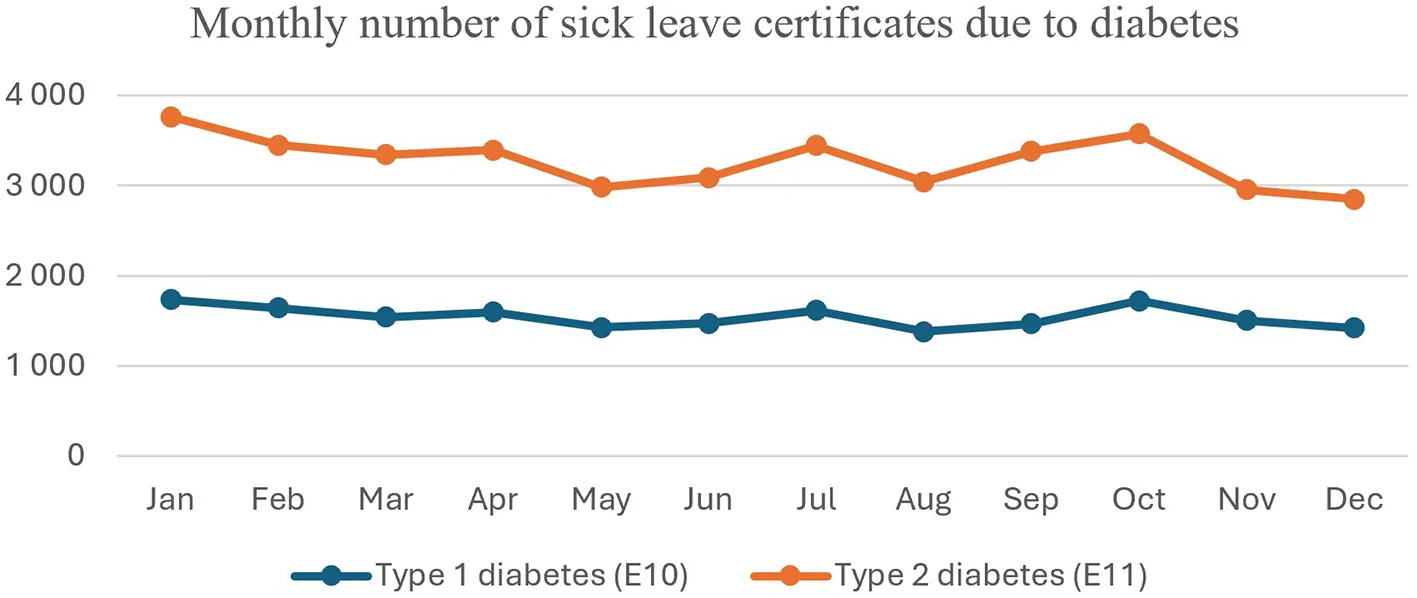
Monthly number of sick leave certificates due to diabetes type 1 (ICD-10: E10) or type 2 (ICD-10: E11), Poland, 2024.

There were differences in sick leave days and sick leave certificate rates by diabetes type, patient age, and sex (Table 2). For type 1 diabetes, overall rates declined between 2020 and 2024. Specifically, the overall rate of sick leave days decreased from 1,190.2 per 100,000 in 2020 to 969.8 per 100,000 in 2024, while the overall rate of certificates decreased slightly from 80.2 to 78.0 per 100,000 (Table 2). Both rates increased consistently with age, reaching their highest values in the 55–59 age group (Table 2). Notably, despite the overall downward trend, certificate rates increased in younger adults (18–34). The relative increase was most noticeable in the youngest group (18–19), where the number of certificates issued for type 1 diabetes increased from 29.5 to 74.4 per 100,000 between 2020 and 2024 (Table 2). Moreover, the 18–19 age group was the only group to show an increase in the overall sick leave days rate over time, with sick leave days due to type 1 diabetes rising from 220.9 per 100,000 in 2020 to 338.3 per 100,000 in 2024.

**Table 2:** Number of sick leave days and sick leave certificates due to diabetes type 1 (ICD-10: E10) or type 2 (ICD-10: E11) per 100,000 population in a given age group, Poland, 2020–2024.

Year	Number of sick leave days due to diabetes per 100,000 population					Number of sick leave certificates due to diabetes per 100,000 population				
	2020	2021	2022	2023	2024	2020	2021	2022	2023	2024
Type 1 diabetes (E10)										
Overall										
**Overall**	**1,190.2**	**987.6**	**937.8**	**967.8**	**969.8**	**80.2**	**69.8**	**69.6**	**74.9**	**78**
18–19	220.9	174.5	266.2	334.3	338.3	29.5	33.3	47.9	65.8	74.4
20–24	606.4	478.7	453.7	412.1	504.1	54.5	50.6	49.9	50.1	59.4
25–29	787.9	663.4	643.2	672.6	709.7	67.8	64.0	70.6	79.0	89.2
30–34	857.0	653.6	605.5	700.9	694.8	68.2	55.6	57.6	67.0	70.1
35–39	1,037.7	831.7	762.9	775.1	736.0	74.4	65.1	63.8	67.9	68.3
40–44	1,190.0	975.4	903.1	898.6	901.0	81.4	69.3	67.7	71.3	73.4
45–49	1,433.5	1,186.6	1,113.2	1,178.4	1,081.2	93.9	79.2	76.8	83.6	80.3
50–54	1,660.6	1,376.0	1,423.6	1,365.2	1,344.1	102.2	88.3	88.6	89.0	92.1
55–59	1,779.5	1,652.9	1,519.6	1,552.1	1,599.2	104.1	96.2	90.9	95.5	98.0
60–64	1,618.6	1,286.3	1,179.1	1,257.4	1,294.8	89.3	70.9	66.0	70.7	73.0
Females										
**Overall**	**746.2**	**537.8**	**530.3**	**527.8**	**545.7**	**54.0**	**42.9**	**45**	**48**	**52.5**
18–19	201.2	107.4	232.4	272.1	304.4	26.5	20.5	42.6	51.9	59.5
20–24	542.6	475.5	387.4	372.9	390.6	47.1	46.5	42.5	45.2	49.5
25–29	743.0	533.4	513.1	454.7	579.4	65.3	57.5	62.7	60.6	81.4
30–34	734.8	548.9	514.3	488.5	532.1	58.2	47.1	49.6	50.6	58.8
35–39	700.9	533.6	547.2	566.9	492.5	51.0	42.7	47.8	54.2	51.9
40–44	794.9	585.8	531.1	506.1	530.6	57.7	45.3	43.3	44.0	47.7
45–49	918.6	591.2	607.8	673.5	637.9	61.8	42.7	46.2	56.9	54.8
50–54	970.9	740.0	714.5	608.1	736.7	62.0	52.3	48.4	47.7	57.7
55–59	1,090.8	798.5	793.0	821.0	774.9	68.0	50.7	51.2	54.5	53.9
60–64	397.4	181.0	232.1	273.8	266.1	25.7	13.4	16.9	17.5	19.7
Males										
**Overall**	**1,634.8**	**1,437.9**	**1,345.3**	**1,407.4**	**1,392.9**	**106.5**	**96.8**	**94.1**	**101.8**	**103.4**
18–19	239.6	238.4	298.4	393.4	370.7	32.3	45.5	53.0	79.1	88.6
20–24	667.4	481.8	517.4	449.8	612.8	61.6	54.6	57.0	54.9	68.9
25–29	831.4	789.3	769.1	883.5	835.5	70.1	70.3	78.3	96.8	96.8
30–34	975.5	755.0	693.9	906.6	852.6	77.8	63.8	65.4	82.9	81.0
35–39	1,366.8	1,122.0	973.2	978.2	972.8	97.3	87.1	79.5	81.3	84.3
40–44	1,578.1	1,357.8	1,268.3	1,284.0	1,264.8	104.7	92.8	91.6	98.2	98.6
45–49	1,944.9	1,777.7	1,615.0	1,679.7	1,521.2	125.8	115.4	107.0	110.1	105.7
50–54	2,360.4	2,021.1	2,142.4	2,132.0	1,958.1	143.0	124.9	129.4	130.8	126.9
55–59	2,504.7	2,552.0	2,282.5	2,317.2	2,461.3	142.1	144.1	132.6	138.4	144.1
60–64	2,994.4	2,533.5	2,242.2	2,355.3	2,435.1	160.9	135.8	121.1	130.0	132.0
Type 2 diabetes (E11)										
Overall										
**Overall**	**1,969**	**1,978.7**	**1,881.2**	**1,948.6**	**1,956.3**	**143.6**	**149.6**	**148.3**	**156.1**	**158.9**
18–19	36.4	19.8	7.4	19.7	12.1	2.9	3.0	1.7	3.5	3.0
20–24	75.0	43.8	62.5	58.7	66.9	7.4	5.7	8.3	7.2	7.0
25–29	147.9	145.6	140.4	133.8	159.1	14.2	15.7	15.2	15.3	18.4
30–34	362.3	342.2	342.4	309.0	344.1	30.5	31.9	33.2	31.8	34.7
35–39	681.5	690.9	648.0	698.3	638.0	55.7	60.9	62.1	66.0	62.7
40–44	1,335.3	1,228.5	1,188.8	1,237.4	1,205.4	103.8	104.2	101.9	110.1	111.4
45–49	2,443.9	2,437.9	2,278.2	2,270.5	2,296.5	185.3	192.2	186.6	191.0	197.9
50–54	3,715.2	3,930.5	3,637.4	3,719.5	3,596.7	278.1	302.1	293.2	303.2	289.4
55–59	5,034.8	5,304.4	4,845.9	5,206.2	5,161.0	357.9	381.1	366.5	396.3	400.0
60–64	4,857.6	4,647.0	4,603.6	4,666.5	4,804.5	326.2	318.6	324.9	334.5	349.9
Females										
**Overall**	**983.1**	**886**	**792.9**	**811.4**	**821.1**	**75.5**	**72.1**	**70.1**	**76.3**	**77.9**
18–19	48.2	32.3	9.5	19.3	16.7	4.6	5.0	1.8	4.3	4.4
20–24	73.3	30.9	41.1	62.0	67.6	7.1	5.0	7.1	8.6	6.8
25–29	134.8	123.8	123.2	103.5	105.1	13.2	13.3	13.7	13.1	14.8
30–34	184.5	231.5	216.0	184.4	217.6	17.7	22.1	22.0	22.3	23.8
35–39	344.7	327.6	377.4	421.6	378.8	28.9	30.8	37.6	44.2	39.7
40–44	752.8	642.4	538.6	552.3	580.7	58.9	57.2	49.5	58.4	61.3
45–49	1,251.9	1,107.7	1,057.7	946.7	1,010.2	98.6	93.0	92.6	93.2	100.7
50–54	2,071.4	1,997.7	1,662.8	1,752.2	1,693.4	160.7	162.9	150.8	160.3	156.0
55–59	3,101.9	2,867.6	2,415.5	2,500.3	2,510.6	225.2	215.8	200.2	217.2	218.8
60–64	1,367.4	1,020.0	940.9	969.0	922.6	101.3	75.9	77.3	82.2	83.0
Males										
**Overall**	**2,956.2**	**3,072.5**	**2,969.5**	**3,084.5**	**3,089.1**	**211.9**	**227.2**	**226.5**	**235.9**	**239.8**
18–19	25.2	7.9	5.4	20.0	7.7	1.4	1.1	1.7	2.7	1.6
20–24	76.6	56.2	83.0	55.4	66.3	7.7	6.4	9.4	5.8	7.2
25–29	160.5	166.6	157.1	163.1	211.3	15.1	17.9	16.5	17.4	21.9
30–34	534.9	449.4	464.9	429.6	466.8	42.9	41.4	44.1	41.0	45.2
35–39	1,010.7	1,044.7	911.7	968.2	890.9	81.9	90.2	85.9	87.2	85.1
40–44	1,907.6	1,803.9	1,827.1	1,910.1	1,819.0	147.9	150.4	153.2	160.8	160.6
45–49	3,627.7	3,758.5	3,490.0	3,583.9	3,573.1	271.4	290.6	279.9	288.1	294.5
50–54	5,382.5	5,890.8	5,639.0	5,712.1	5,520.5	397.2	443.3	437.6	448.0	424.2
55–59	7,070.0	7,868.3	7,397.5	8,038.3	7,930.6	497.6	554.9	541.1	583.7	589.2
60–64	8,789.9	8,739.6	8,715.4	8,793.6	9,107.6	579.5	592.5	602.9	616.2	645.8

Statistically significant values are bolded.

Across all years, the burden of type 2 diabetes was higher than that of type 1 diabetes, both in the number of sick leave days and certificates per 100,000 population. The overall rate of sickness absence due to type 2 diabetes also decreased slightly, from 1,969.0 per 100,000 in 2020 to 1,956.3 per 100,000 in 2024 (Table 2). However, the rate of sick leave certificates issued due to type 2 diabetes increased from 143.6 to 158.9 per 100,000 during the same period (Table 2). For type 2 diabetes, both rates also increased with age and reached the highest levels in the 55–59 age group, but the increase was much sharper, with very low overall rates in ages 18–29 and a steep increase after age 40 (Table 2).

For both type 1 and type 2 diabetes, men had consistently higher rates of sick leave days and sick leave certificates than women across all years and age groups (Table 2). The highest rates of sick leave days and certificates were observed in the 60–64 age group for men and the 55–59 age group for women for both diabetes types (Table 2). Among women, a substantial drop in both rates was observed from ages 55–59 to 60–64, such that women aged 60–64 had considerably lower rates of sick leave days and certificates than men of the same age (Table 2). For example, in 2024, women showed 266.1 sick leave days per 100,000 and 19.7 certificates per 100,000 for type 1 diabetes, and 922.6 sick leave days per 100,000 and 83.0 certificates per 100,000 for type 2 diabetes. Meanwhile, men aged 60–64 showed 2,435.1 sick leave days per 100,000 and 132.0 certificates per 100,000 for type 1 diabetes, and 9,107.6 sick leave days per 100,000 and 645.8 certificates per 100,000 for type 2 diabetes (Table 2).

There were county-level geographical differences in the distributions of sick leave days and certificates per 10,000 population between type 1 and type 2 diabetes in 2024 (Figure 2). For type 1 diabetes, both the rates of sickness absence and sick leave certificates were relatively evenly distributed across counties, with small zones of higher rates in the central and northwestern regions and generally lower rates in many eastern counties, but no obvious large area clustering (Figure 2). For type 2 diabetes, the distributions were more heterogeneous and showed clearer clustering, with higher rates mainly in the southern and southwestern regions and parts of the central region, and lower rates in the northern and eastern regions (Figure 2). In general, the distribution of sickness absence rates was similar to the distribution of certificate rates for both diabetes types (Figure 2).

**Figure 2:**
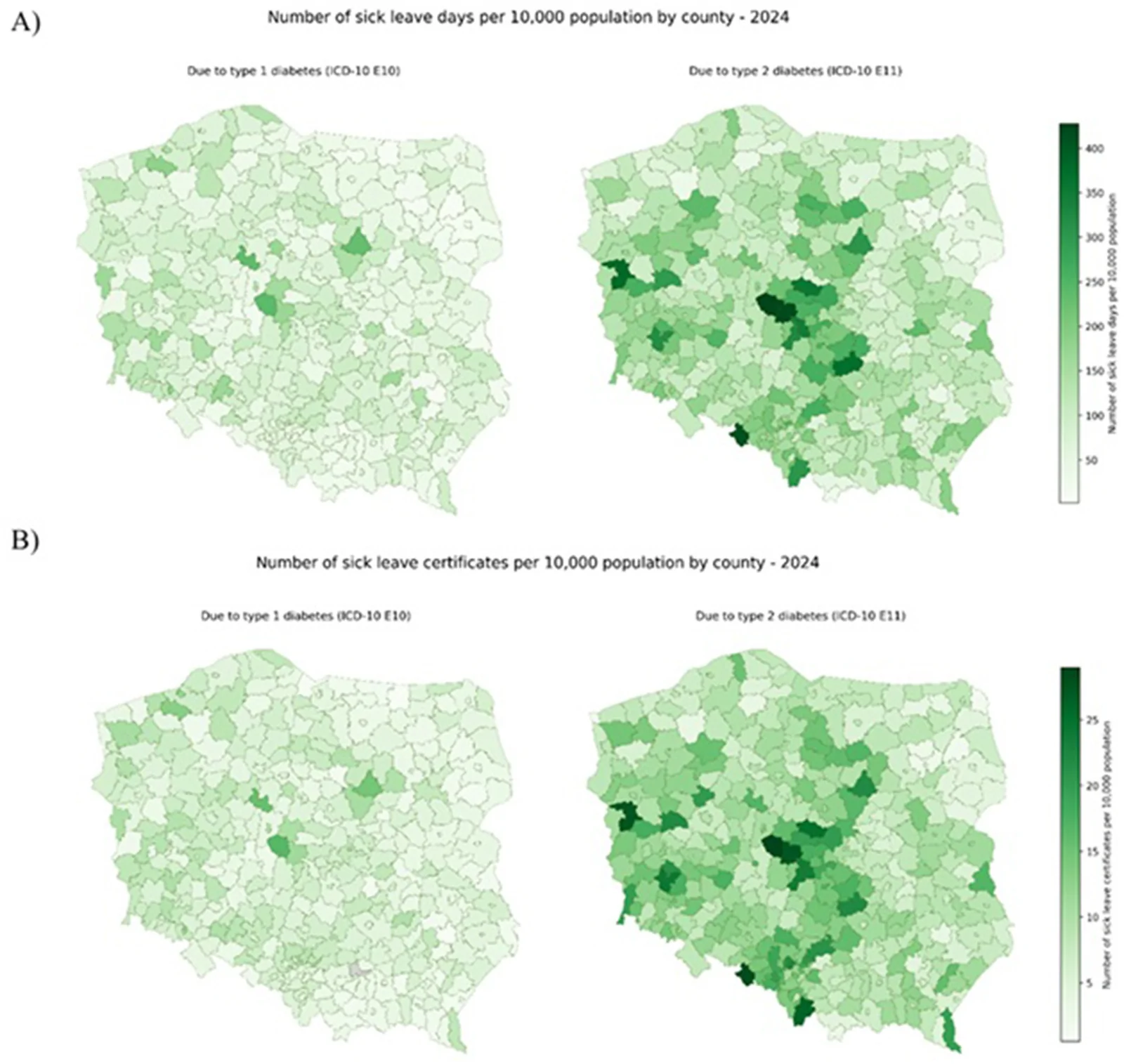
County-level distribution of the number of **(A)** sick leave days and **(B)** certificates due to type 1 (ICD-10: E10) and type 2 (ICD-10: E11) diabetes per 10,000 population, Poland, 2024.

The cumulative average length of sickness absence per year (all medical certificates issued to a particular patient within 1 year) due to type 1 diabetes was: 33.17 days in 2020 (M:35.62; F: 28.76), 29.93 days in 2021 (M:32.8; F:24.12), 26.77 days in 2022 (M:29.34; F:21.81), 25.37 days in 2023 (M:28.12; F:20.06) and 24.14 days in 2024 (M:27.26; F:18.63). The cumulative average length of sickness absence per year due to type 2 diabetes was: 29.89 days in 2020 (M:30.12; F:25.65), 26.64 days in 2021 (M:28.14; F:22.45), 24.18 days in 2022 (M:25.97; F:19.09), 23.3 days in 2023 (M:25.54; F:17.38) and 23.03 days in 2024 (M:25.22; F:17.3).

The average duration of sick leave per certificate decreased for both diabetes types between 2020 and 2024 (Table 3). The overall average duration decreased from 14.83 (2020) to 12.44 days (2024) for type 1 diabetes and from 13.71 (2020) to 12.31 days (2024) for type 2 diabetes (Table 3). The average duration of sick leave was slightly longer for type 1 than for type 2 diabetes across most years and age groups. The average duration of sick leave increased steadily with age, reaching the longest durations in the 60–64 age group for both diabetes types (Table 3). Men had generally longer average durations of sick leave compared with women for both diabetes types, with the difference being more pronounced for type 1 diabetes (Table 3).

**Table 3:** Sick leave duration [days] per certificate ratio due to type 1 or type 2 diabetes, 2020–2024, Poland.

Year	2020	2021	2022	2023	2024	2020	2021	2022	2023	2024	2020	2021	2022	2023	2024
	Overall					Males					Females				
Average duration of sick leave [days] per sick leave certificate due to type 1 diabetes (E10)															
**Overall**	14.83	14.14	13.48	12.92	12.44	15.35	14.86	14.29	13.83	13.47	13.82	12.52	11.78	10.99	10.39
18–19	7.49	5.24	5.55	5.08	4.55	7.42	5.24	5.63	4.98	4.19	7.58	5.25	5.45	5.24	5.12
20–24	11.13	9.46	9.09	8.22	8.49	10.84	8.83	9.07	8.20	8.90	11.53	10.23	9.11	8.24	7.89
25–29	11.63	10.36	9.11	8.51	7.96	11.86	11.22	9.82	9.12	8.64	11.37	9.28	8.19	7.50	7.12
30–34	12.57	11.76	10.52	10.46	9.91	12.53	11.83	10.62	10.94	10.52	12.62	11.66	10.38	9.65	9.04
35–39	13.95	12.77	11.95	11.41	10.77	14.05	12.89	12.25	12.03	11.54	13.74	12.51	11.45	10.45	9.50
40–44	14.62	14.08	13.34	12.60	12.28	15.07	14.63	13.85	13.08	12.82	13.78	12.93	12.26	11.51	11.13
45–49	15.27	14.99	14.50	14.10	13.46	15.46	15.41	15.09	15.25	14.40	14.87	13.86	13.14	11.84	11.65
50–54	16.24	15.58	16.06	15.34	14.60	16.50	16.18	16.55	16.30	15.43	15.65	14.16	14.77	12.76	12.78
55–59	17.10	17.19	16.72	16.25	16.32	17.63	17.71	17.22	16.74	17.08	16.05	15.76	15.49	15.07	14.38
60–64	18.13	18.15	17.87	17.80	17.74	18.61	18.66	18.52	18.12	18.45	15.46	13.55	13.70	15.67	13.47
Average duration of sick leave [days] per sick leave certificate due to type 2 diabetes (E11)															
**Overall**	13.71	13.23	12.68	12.48	12.31	13.95	13.52	13.11	13.08	12.88	13.02	12.29	11.31	10.64	10.54
18–19	12.48	6.52	4.25	5.60	4.05	18.60	7.00	3.17	7.30	4.83	10.56	6.41	5.33	4.47	3.75
20–24	10.12	7.71	7.51	8.19	9.52	9.88	8.85	8.80	9.53	9.18	10.38	6.20	5.75	7.24	9.88
25–29	10.45	9.30	9.26	8.76	8.64	10.66	9.30	9.50	9.39	9.63	10.20	9.30	8.97	7.91	7.12
30–34	11.88	10.72	10.30	9.71	9.92	12.47	10.85	10.54	10.48	10.32	10.41	10.47	9.81	8.25	9.13
35–39	12.23	11.34	10.44	10.59	10.18	12.34	11.58	10.61	11.10	10.46	11.92	10.62	10.04	9.55	9.54
40–44	12.87	11.79	11.67	11.24	10.82	12.90	12.00	11.92	11.88	11.33	12.79	11.24	10.87	9.45	9.47
45–49	13.19	12.69	12.21	11.89	11.60	13.37	12.93	12.47	12.44	12.13	12.70	11.91	11.43	10.16	10.03
50–54	13.36	13.01	12.41	12.27	12.43	13.55	13.29	12.89	12.75	13.01	12.89	12.26	11.03	10.93	10.85
55–59	14.07	13.92	13.22	13.14	12.90	14.21	14.18	13.67	13.77	13.46	13.77	13.29	12.06	11.51	11.47
60–64	14.89	14.58	14.17	13.95	13.73	15.17	14.75	14.45	14.27	14.10	13.49	13.44	12.18	11.79	11.11

Overall, type 1 diabetes accounted for more medical certificates granting entitlement to rehabilitation benefit than type 2 diabetes (Table 4). However, the difference narrowed over time, and the totals became more comparable in 2022–2024 than in 2020–2021. Between 2020 and 2024, there was a slight decrease in the overall number of certificates issued for type 1 diabetes (−2.9%) and a moderate increase for type 2 diabetes (+16.7%) (Table 4). For both type 1 and type 2 diabetes, rehabilitation benefit certificates were issued predominantly to men (Table 4). Across all years, approximately three-quarters of rehabilitation benefit certificates due to type 1 diabetes and approximately four-fifths of those due to type 2 diabetes were issued to men (Table 4).

**Table 4:** Number of medical certificates granting entitlement to rehabilitation benefit due to diabetes type 1 or 2, Poland, 2020–2024.

	2020			2021			2022			2023			2024			% change 2024–2020		
	T	M	F	T	M	F	T	M	F	T	M	F	T	M	F	T	M	F
Number of medical certificates granting entitlement to rehabilitation benefit due to type 1 diabetes (E10)																		
Total	750	562	185	695	517	177	731	543	187	701	541	159	728	584	144	−2.9	3.9	−22.2
Dolnoślaskie	62	42	20	55	45	10	30	22	8	56	40	16	64	49	15	3.2	16.7	−25.0
Kujawsko-Pomorskie	30	26	4	29	20	9	38	29	9	47	36	11	60	50	10	100.0	92.3	150.0
Lubelskie	36	28	8	23	19	4	39	28	11	28	27	0	33	29	4	−8.3	3.6	−50.0
Lubuskie	26	20	6	13	12	0	13	12	0	17	12	5	15	11	4	−42.3	−45.0	−33.3
Łódzkie	129	92	36	109	76	32	127	104	23	97	74	23	96	76	20	−25.6	−17.4	−44.4
Małopolskie	26	19	7	30	25	5	39	33	6	38	34	4	31	24	7	19.2	26.3	0.0
Mazowieckie	76	56	20	76	55	21	72	53	19	76	58	18	84	72	12	10.5	28.6	−40.0
Opolskie	36	28	7	28	21	7	27	25	0	24	20	4	18	18	0	−50.0	−35.7	−100.0
Podkarpackie	18	12	6	16	16	0	17	12	5	11	7	4	20	14	6	11.1	16.7	0.0
Podlaskie	10	8	0	9	9	0	4	3	0	0	0	0	6	6	0	−40.0	−25.0	–
Pomorskie	57	45	12	56	37	19	69	49	20	67	54	13	51	44	7	−10.5	−2.2	−41.7
Slaskie	86	66	19	109	76	33	106	79	26	94	66	27	94	66	28	9.3	0.0	47.4
Swietokrzyskie	32	26	6	25	19	6	39	25	14	24	20	4	43	37	6	34.4	42.3	0.0
Warmińsko-Mazurskie	32	26	6	30	23	7	19	13	6	13	12	0	28	23	5	−12.5	−11.5	−16.7
Wielkopolskie	40	25	15	32	17	15	50	30	20	49	40	9	39	32	7	−2.5	28.0	−53.3
Zachodniopomorskie	54	43	11	55	47	8	42	26	16	59	40	19	46	33	13	−14.8	−23.3	18.2
Number of medical certificates granting entitlement to rehabilitation benefit due to type 2 diabetes (E11)																		
Total	594	489	103	576	449	124	662	520	138	745	585	159	693	581	108	16.7	18.8	4.9
Dolnoślaskie	40	30	10	54	46	8	48	40	8	58	48	10	53	49	4	32.5	63.3	−60.0
Kujawsko-Pomorskie	32	25	7	37	24	13	37	24	13	38	29	9	37	27	10	15.6	8.0	42.9
Lubelskie	35	27	8	25	22	3	25	21	4	26	24	0	28	25	3	−20.0	−7.4	−62.5
Lubuskie	8	7	0	18	14	3	29	21	8	36	27	9	40	32	8	400.0	357.1	–
Łódzkie	59	50	8	45	40	5	50	37	13	47	36	11	59	47	11	0.0	−6.0	37.5
Małopolskie	37	33	4	50	41	9	78	63	15	63	54	9	51	47	4	37.8	42.4	0.0
Mazowieckie	48	39	9	46	36	10	44	31	13	69	55	14	76	63	12	58.3	61.5	33.3
Opolskie	22	16	5	10	10	0	18	16	0	15	14	0	29	25	4	31.8	56.3	−20.0
Podkarpackie	43	38	5	44	38	6	37	31	6	37	28	9	33	28	5	−23.3	−26.3	0.0
Podlaskie	16	13	3	14	12	0	6	5	0	14	10	4	15	13	0	−6.3	0.0	−100.0
Pomorskie	26	23	3	21	15	6	20	17	3	20	14	6	22	19	0	−15.4	−17.4	−100.0
Slaskie	106	89	17	100	68	30	147	115	30	167	125	41	142	126	15	34.0	41.6	−11.8
Swietokrzyskie	13	11	0	10	8	0	13	11	0	12	11	0	10	6	4	−23.1	−45.5	–
Warmińsko-Mazurskie	16	15	0	17	17	0	20	18	0	28	23	5	26	18	8	62.5	20.0	–
Wielkopolskie	74	57	17	78	54	24	77	58	19	104	80	24	58	45	13	−21.6	−21.1	−23.5
Zachodniopomorskie	19	16	3	7	4	3	13	12	–	11	7	4	14	11	3	−26.3	−31.3	0.0

T, total; M, males; F, females.

There were regional variations in the number of medical certificates granting entitlement to rehabilitation benefit for both type 1 and type 2 diabetes across voivodeships (Table 4). For type 1 diabetes, the highest counts were generally observed in Łódzkie, Slaskie, and Mazowieckie voivodeships, while the lowest counts were observed in Podlaskie, Podkarpackie, and Lubuskie voivodeships (Table 4). For type 2 diabetes, the highest counts were observed in Slaskie and Wielkopolskie, as well as Łódzkie and Mazowieckie voivodeships in later years, and the lowest counts were typically observed in Swietokrzyskie, Zachodniopomorskie, and Podlaskie voivodeships (Table 4). Changes in counts from 2020 to 2024 also varied by region. For type 1 diabetes, certificate counts decreased in 9 of 16 voivodeships, with the largest increase observed in Kujawsko-Pomorskie (+100.0%) and the largest decrease in Opolskie (−50.0%) (Table 4). For type 2 diabetes, increases were observed in half of voivodeships (8 of 16), with the largest increase in Lubuskie (+400.0%), and the largest decrease in Zachodniopomorskie (−26.3%) (Table 4).

Yearly social insurance expenditures increased between 2020 and 2024 for both type 1 and type 2 diabetes (Table 5). Expenditures were substantially higher for sickness absence than for rehabilitation benefits. For type 1 diabetes, total expenditures increased by 16.1%, from 23,841.8 thousand PLN (approximately 5.37 million EUR) to 27,685.8 thousand PLN (approximately 6.44 million EUR) (Table 5). For type 2 diabetes, expenditures were consistently higher, increasing by 39.8% from 46,228.8 thousand PLN (approximately 10.41 million EUR) to 64,627.3 thousand PLN (approximately 15.03 million EUR) (Table 5). Most sickness absence expenditures were attributable to men, accounting for about 69%−73% of type 1 diabetes costs and 76%−79% of type 2 diabetes costs across the study period (Table 5). Expenditures on rehabilitation benefits also increased over time. For type 1 diabetes, rehabilitation benefit costs increased by 38.3%, from 8,454.7 thousand PLN (approximately 1.90 million EUR) to 11,693.5 thousand PLN (approximately 2.72 million EUR), while for type 2 diabetes, they increased by 69.4%, from 6,569.7 thousand PLN (approximately 1.48 million EUR) to 11,131.3 PLN thousand PLN (approximately 2.59 million EUR) (Table 5).

**Table 5:** Social insurance expenditures (in PLN thousand) on sickness absence and rehabilitation benefits in 2020–2024, Poland.

	2020	2021	2022	2023	2024	% change 2024–2020	% change 2024–2023
Social insurance expenditures (in PLN thousand) on sickness absence due to type 1 diabetes (E10)							
Total	23,841.8	21,455.2	21,714.3	23,159.6	27,685.8	16.1	19.5
Males	16,540.2	15,648.7	15,428.9	16,868.0	20,054.8	29.5	15.4
% of total costs	69.4	72.9	71.1	72.8	72.4	15.0	19.2
Dolnoślaskie	1,931.4	1,887.3	1,983.1	2,166.3	2,500.9	25.4	34.7
Kujawsko-Pomorskie	1,483.4	1,400.8	1,250.4	1,431.0	1,705.7	18.2	22.7
Lubelskie	1,076.4	924.9	992.6	1,001.5	1,349.4	8.6	19.4
Lubuskie	787.1	664.6	707.8	758.4	930.6	19.4	21.2
Łódzkie	2,083.5	1,852.6	1,962.1	1,894.0	2,261.8	18.2	19.5
Małopolskie	1,389.2	1,291.4	1,187.7	1,369.0	1,659.3	45.2	48.7
Mazowieckie	3,033.7	2,724.8	2,824.5	3,001.6	3,587.3	1.6	25.7
Opolskie	535.6	499.6	510.7	523.0	777.7	−15.9	17.2
Podkarpackie	1,391.8	1,263.9	1,136.9	1,125.0	1,414.5	0.8	22.0
Podlaskie	413.2	307.4	364.7	296.3	347.3	12.7	12.3
Pomorskie	1,649.7	1,539.9	1,422.4	1,363.6	1,663.6	−0.9	−1.5
Slaskie	2,787.2	2,548.1	2,621.7	2,797.7	3,140.7	5.7	13.5
Swietokrzyskie	1,125.0	912.9	973.3	1,132.4	1,114.9	20.4	19.9
Warmińsko-Mazurskie	793.3	702.7	660.9	738.7	838.5	55.2	30.3
Wielkopolskie	2,363.6	1,900.3	2,000.7	2,372.4	2,844.7	16.1	19.5
Zachodniopomorskie	997.8	1,034.2	1,114.7	1,188.5	1,548.9	29.5	15.4
Social insurance expenditures (in PLN thousand) on sickness absence due to type 2 diabetes (E11)							
Total	46,228.8	51,212.4	50,215.8	54,983.3	64,627.3	39.8	17.5
Males	35,101.5	39,756.2	39,867.2	43,732.9	51,186.5	44.6	12.0
% of total costs	75.9	77.6	79.4	79.5	79.2	35.6	11.8
Dolnoślaskie	3,555.8	4,081.9	4,030.1	4,592.7	5,143.0	45.6	30.3
Kujawsko-Pomorskie	2,415.3	2,830.9	2,672.6	2,929.6	3,274.3	58.4	15.5
Lubelskie	1,980.0	2,198.1	2,330.8	2,212.9	2,882.6	19.6	21.2
Lubuskie	1,362.5	1,472.4	1,636.3	1,867.9	2,157.8	41.5	17.9
Łódzkie	5,913.8	5,958.1	5,654.4	5,833.9	7,072.9	45.4	21.1
Małopolskie	3,479.3	3,817.2	3,818.7	4,175.3	4,923.3	79.4	18.5
Mazowieckie	4,953.0	5,429.2	5,174.5	5,948.7	7,201.9	3.9	4.9
Opolskie	1,040.2	1,279.1	1,160.1	1,575.1	1,866.6	20.3	2.7
Podkarpackie	3,242.1	3,265.9	3,084.3	3,211.6	3,369.8	58.8	20.1
Podlaskie	719.3	865.1	761.7	842.8	865.5	56.6	25.5
Pomorskie	1,887.4	2,037.0	2,496.5	2,496.1	2,997.7	19.6	16.5
Slaskie	6,336.4	7,580.7	7,264.7	7,907.6	9,921.2	59.1	20.8
Swietokrzyskie	1,872.3	1,840.3	1,690.6	1,922.4	2,239.9	36.7	14.2
Warmińsko-Mazurskie	1,289.6	1,530.5	1,705.0	1,698.6	2,051.7	52.7	3.2
Wielkopolskie	4,890.1	5,352.8	5,227.3	5,857.1	6,686.4	39.8	17.5
Zachodniopomorskie	1,291.7	1,673.4	1,508.1	1,911.1	1,972.6	44.6	12.0
Social insurance expenditures (in PLN thousand) on rehabilitation benefits due to type 1 diabetes (E10)							
Total	8,454.7	8,177.2	8,641.2	9,633.6	11,693.5	38.3	21.4
Dolnoślaskie	683.4	644.3	400.4	769.6	1,028.0	50.4	33.6
Kujawsko-Pomorskie	341.7	339.7	404.7	645.9	963.7	182.0	49.2
Lubelskie	396.8	269.5	403.5	384.8	530.1	33.6	37.8
Lubuskie	330.7	152.3	130.7	233.6	240.9	−27.2	3.1
Łódzkie	1,488.1	1,288.7	1,201.4	1,333.0	1,542.0	3.6	15.7
Małopolskie	297.6	351.5	418.8	522.2	497.9	67.3	−4.7
Mazowieckie	837.8	890.4	1,488.9	1,044.4	1,349.2	61.0	29.2
Opolskie	396.8	328.0	272.1	329.8	289.1	−27.1	−12.3
Podkarpackie	209.4	187.4	201.8	151.2	321.2	53.4	112.4
Podlaskie	110.2	105.4	47.1	13.7	96.4	−12.5	603.6
Pomorskie	628.3	656.1	666.2	920.8	819.2	30.4	−11.0
Slaskie	959.0	1,277.0	1,186.8	1,291.8	1,509.9	57.4	16.9
Swietokrzyskie	374.8	304.6	342.1	329.8	690.7	84.3	109.4
Warmińsko-Mazurskie	352.7	351.5	198.0	178.7	449.7	27.5	151.7
Wielkopolskie	440.9	374.9	841.8	673.4	626.4	42.1	−7.0
Zachodniopomorskie	606.3	656.1	437.0	810.8	738.9	21.9	−8.9
Social insurance expenditures (in PLN thousand) on rehabilitation benefits due to type 2 diabetes (E11)							
Total	6,569.7	6,888.6	8,087.8	10,375.7	11,131.3	69.4	7.3
Dolnoślaskie	451.9	632.6	620.0	797.1	851.3	88.4	6.8
Kujawsko-Pomorskie	352.7	445.2	385.4	522.2	594.3	68.5	13.8
Lubelskie	385.8	292.9	279.3	357.3	449.7	16.6	25.9
Lubuskie	88.2	246.0	291.6	508.5	642.5	628.5	26.4
Łódzkie	650.4	538.9	473.0	700.9	947.7	45.7	35.2
Małopolskie	407.9	585.8	870.7	879.5	819.2	100.8	−6.9
Mazowieckie	529.1	538.9	897.4	948.2	1,220.7	130.7	28.7
Opolskie	242.5	117.2	181.4	206.1	465.8	92.1	126.0
Podkarpackie	474.0	515.5	439.2	508.5	530.1	11.8	4.2
Podlaskie	176.4	164.0	70.7	192.4	240.9	36.6	25.2
Pomorskie	286.6	246.0	193.1	302.3	353.4	23.3	16.9
Slaskie	1 168.4	1,171.5	1,596.7	2,322.5	2,280.9	95.2	−1.8
Swietokrzyskie	143.3	175.7	149.1	164.9	160.6	12.1	−2.6
Warmińsko-Mazurskie	176.4	199.2	208.4	384.8	417.6	136.7	8.5
Wielkopolskie	815.7	913.8	1 296.4	1,429.2	931.6	14.2	−34.8
Zachodniopomorskie	220.5	105.4	135.3	151.2	224.9	2.0	48.7

There were regional differences in social insurance expenditures related to both sickness absence and rehabilitation benefits across voivodeships (Table 5). For both type 1 and type 2 diabetes, spending was consistently highest in Slaskie, Mazowieckie, Łódzkie, and Wielkopolskie voivodeships and lowest in Podlaskie and Opolskie voivodeships (Table 5). Between 2020 and 2024, expenditures due to sickness absence for type 1 diabetes increased the most in Warmińsko-Mazurskie (+55.2%) and Małopolskie (+45.2%) and decreased in Opolskie (−15.9%) and Pomorskie (−0.9%) voivodeships only (Table 5). For type 2 diabetes, the largest increases were observed in Małopolskie (+79.4%) and Slaskie (+59.1%), with no decrease observed in any voivodeships throughout this period (Table 5).

For expenditures related to rehabilitation benefits, Slaskie, Łódzkie, Mazowieckie, and Wielkopolskie had the highest spending for both diabetes types (Table 5). Podlaskie had the lowest expenditures for type 1 diabetes, whereas for type 2 diabetes, the lowest-cost voivodeships varied from year to year, most often including Swietokrzyskie and Zachodniopomorskie (Table 5). Between 2020 and 2024, the largest increases in spending on rehabilitation benefits for type 1 diabetes were in Kujawsko-Pomorskie (+182.0%) and Swietokrzyskie (+84.3%), while the largest decreases were in Lubuskie (−27.2%) and Opolskie (−27.1%) (Table 5). For type 2 diabetes, the largest increases were observed in Lubuskie (+628.5%) and Warmińsko-Mazurskie (+136.7%). No decreases were observed from 2020 to 2024, but the largest decreases from 2023 to 2024 occurred in Wielkopolskie (−34.8%) and Małopolskie (−6.9%) voivodeships (Table 5).

## 4 Discussion

This study provides a comprehensive epidemiological characterization of diabetes-related sickness absence in Poland between 2020 and 2024, based on nationwide data from the Social Insurance Institution (ZUS) covering 16.6 million insured adults. The results show that, despite an overall reduction in sick leave days for both type 1 and type 2 diabetes, the burden of diabetes-related work incapacity remained substantial throughout the study period, and several subgroup-specific and regional trends emerged with important implications for public health policy and clinical practice.

The overall decline in sick leave days for type 1 and type 2 diabetes between 2020 and 2024 is a relevant finding given the generally increasing prevalence of both diabetes types in Poland. The number of Poles living with diagnosed diabetes has grown steadily, rising from approximately 2.11 million in 2014 to over 3 million at present, with projections indicating a further rise to 4.2 million by 2030 (4, 8). However, the study period encompassed both the COVID-19 pandemic and the post-pandemic period, which should be considered when interpreting temporal trends. Changes in healthcare accessibility, medical certification practices, remote working, labor market conditions, and other pandemic-related factors may have contributed to the observed sickness absence patterns.

The number of sick leave certificates issued for type 2 diabetes increased from 37,564 in 2020 to 39,285 in 2024, a 4.6% increase, while the total number of sick leave days declined over the same period. This divergence between the number of certificates and the total number of absence days indicates shorter average sickness absence per certificate. The underlying factors cannot be determined from the present aggregate data and may involve changes in clinical, occupational, or administrative factors. A similar pattern—with diabetes contributing substantially to both absenteeism and productivity loss in the working-age population—has been documented in other European countries. A Swedish registry-based study involving over 413,000 people with type 2 diabetes and 4.9 million person-years of follow-up demonstrated that approximately 47% of individuals with type 2 diabetes had any sickness absence, compared with 26% of matched controls, and that diabetes-related complications were the key driver of absence days, with stroke adding 102 days, end-stage renal disease 70 days, and severe vision loss 56 days per year (15). A recent integrative review confirmed that diabetes mellitus is associated with substantial productivity losses, increased absenteeism, presenteeism, and early retirement, with occupational factors such as shift work, long working hours, and chronic stress acting as additional determinants of the disease burden (25).

For type 1 diabetes, overall rates declined between 2020 and 2024. However, certificate rates increased among younger adults aged 18–34, with the increase most pronounced in the 18–19 age group, which was also the only age group to show an increase in the sick leave days rate over time. This finding suggests a rising sickness absence burden due to type 1 diabetes in the youngest adults, despite an overall reduction at the population level. The growing impact of type 1 diabetes on young adults is consistent with international epidemiological data indicating that the global burden of type 1 diabetes in adolescents and young adults has been increasing. Trend analyses from high-income countries have shown nearly universal increases in the incidence and prevalence of type 1 diabetes among adolescents and young adults between 1990 and 2019 (3). An additional contribution from the COVID-19 pandemic cannot be excluded: data from Czechia demonstrated a significant upward inflection in the type 1 diabetes incidence curve during the pandemic years 2020–2021 (26), and a recent Swedish population-based study reported increases in type 1 diabetes incidence of approximately 12% in 2021 and 9% in 2022 compared with pre-pandemic years, particularly among young adult men (27). Furthermore, growing evidence challenges the historical view that type 1 diabetes is primarily a childhood-onset disease, with recent data from a California healthcare database estimating the annual incidence in adults aged 20–45 years at 30.1 per 100,000 (28). The increasing sickness absence rates in the 18–19 age group in Poland may thus reflect both the broader trend of rising type 1 diabetes incidence in young adults and a pandemic-related acceleration of disease onset in individuals who were already at advanced preclinical stages.

For both diabetes types, men had consistently higher rates of sick leave days and certificates than women across all years and age groups. For both type 1 and type 2 diabetes, women showed a considerable drop in both sick leave days and certificate rates between the 55–59 and 60–64 age groups, while men showed no comparable drop and maintained high rates in the 60–64 age group. This difference is most likely related to the statutory retirement age in Poland, where women retire at 60 and men at 65. Once women reach the retirement age of 60, they leave the insured workforce, and their sickness absence events are no longer captured by the ZUS sick leave registry, which only covers persons in employment subject to social insurance contributions. This structural effect is inherent to registry-based sickness absence studies and has been observed in other settings where differential retirement ages apply. A Danish retrospective cohort study with up to 17 years of follow-up showed that people with both type 1 and type 2 diabetes had significantly higher hazard ratios for sickness absence and a higher risk of disability pension, further illustrating how diabetes affects labor market participation, particularly among older workers (16). The divergence in sickness absence rates between men and women in the 60–64 age group, therefore, primarily reflects the differential impact of the statutory retirement age rather than a true biological sex difference in diabetes-related morbidity at that age.

Men generally had longer average durations of sick leave than women for both diabetes types, and this difference was more pronounced for type 1 diabetes. This finding may reflect a higher burden of complications and comorbidities among men, higher occupational demands, or differences in how sick leave is issued and used in men compared with women. People with diabetes, particularly those with longer disease duration, are known to experience substantially higher rates of comorbid conditions, including cardiovascular disease, chronic kidney disease, and neuropathy. Studies from Denmark have shown that both male sex and longer disease duration are associated with a higher risk of disability pension among persons with diabetes, which is consistent with the pattern of longer per-certificate absence observed among men in the present study (16). It is also recognized in the broader sickness absence literature that, for many diagnostic categories, socioeconomic, occupational, and sex-related factors independently contribute to absence duration.

In 2024, patterns of diabetes-related sickness absence in Poland showed regional variation, with type 1 diabetes displaying relatively even distribution and type 2 diabetes showing clearer geographic clustering. Higher rates were observed mainly in the country's southern and central areas, while many northern and eastern regions recorded lower values. These differences broadly align with national trends in diabetes prevalence, service use, and population characteristics. The consistency between regional sickness absence, rehabilitation benefits, and social insurance spending highlights how geographic health disparities can shape the broader economic burden of chronic disease. Such patterns mirror international findings linking social, demographic, and environmental factors to uneven diabetes impacts across regions.

The average number of sickness absence days per certificate increased with age for both diabetes types and was highest in the 60–64 age group, consistent with a greater burden of complications and comorbidity among older adults. International evidence (15, 29) shows that people with longer diabetes duration and diabetes-related complications such as stroke, end-stage renal disease, severe vision loss, and cardiovascular disease accumulate substantially more absence days, which aligns with the age-related gradient in absence days per certificate observed in this study.

The number of rehabilitation benefit certificates decreased slightly between 2020 and 2024 for type 1 diabetes, but increased for type 2 diabetes—by 16.7% overall—indicating a growing contribution of type 2 diabetes to longer-term incapacity for work. In the Polish social insurance system, rehabilitation benefits are granted to persons who have exhausted their entitlement to sickness allowance (up to 182 days) but remain incapable of work, provided that further treatment or rehabilitation is deemed capable of restoring their earning capacity. The increase in rehabilitation benefit certificates for type 2 diabetes is consistent with the broader global trend of rising type 2 diabetes prevalence and its increasing contribution to disability and work incapacity. A Swedish study based on 20 years of linked registry data demonstrated that the long-term effects of type 2 diabetes complications—particularly cardiovascular events, renal disease, and vision impairment—persist in terms of sickness absence for many years after the complication onset, supporting the clinical and policy relevance of monitoring the rehabilitation benefit trajectory (15).

Rehabilitation benefit certificates were issued predominantly to men for both diabetes types, with approximately three-quarters of type 1 and approximately four-fifths of type 2 diabetes rehabilitation certificates issued to men throughout the study period. The higher burden observed among men, particularly in older age groups, should be interpreted in the context of the sex-specific statutory retirement age in Poland (60 years for women and 65 years for men). The marked decline in sickness absence rates among women aged 60–64 years likely reflects, at least in part, the smaller proportion of women remaining in the population eligible for sickness absence benefits after reaching statutory retirement age. The male predominance in long-term work incapacity due to diabetes is consistent with findings from the Danish cohort study, which reported higher hazard ratios for disability pension among men with type 2 diabetes compared with women, and is also consistent with the overall pattern of men having higher rates and longer durations of sick leave observed in the present study (16).

Yearly social insurance expenditures on diabetes-related sickness absence and rehabilitation benefits increased between 2020 and 2024 for both diabetes types. For type 1 diabetes, total expenditures rose by 16.1%, from approximately 5.37 million EUR to 6.44 million EUR. For type 2 diabetes, expenditures were consistently higher, increasing by 39.8% from approximately 10.41 million EUR to 15.03 million EUR. Most sickness absence expenditures were attributable to men, which aligns with results showing that men had higher rates of sick leave days and certificates and, in general, longer average durations per certificate than women. The growing expenditure on type 2 diabetes-related work incapacity is particularly concerning in the context of the projected further increase in the number of people with diabetes in Poland. The economic burden of diabetes extends well beyond these social insurance figures: a US analysis estimated total diabetes-related costs of USD 412.9 billion in 2022, with indirect costs—including reduced employment due to disability, presenteeism, and premature mortality—constituting a substantial component (6). Studies from Germany have projected that productivity-adjusted life years lost due to type 2 diabetes will increase from 12.06 million in 2020 to 15.39 million in 2040, reflecting the long-term trajectory of this burden across European populations (13).

In interpreting expenditure trends, it should be noted that in Poland the amount of sickness absence benefit is linked to the insured person's prior earnings, based on the average monthly salary from the 12 months preceding the onset of incapacity for work, while rehabilitation benefit is calculated from the sickness benefit base and may additionally be subject to quarterly indexation. Therefore, part of the increase in expenditures observed between 2020 and 2024 may reflect the benefit calculation mechanism itself, as well as broader macroeconomic factors, including wage growth and inflation, rather than exclusively a worsening of diabetes-related work incapacity. This is particularly relevant given the substantial inflation recorded in Poland during the study period. Consequently, the nominal increase in expenditures should be interpreted with caution, as the real increase appears smaller for type 2 diabetes and may not be present for type 1 diabetes after adjustment for inflation.

Total social insurance spending for diabetes varied between voivodeships, with Slaskie, Mazowieckie, Łódzkie, and Wielkopolskie showing the highest absolute expenditures, which also coincided with the largest numbers of certificates. These differences largely reflect the greater population size and employment density in these regions, which together account for a substantial share of Poland's insured workforce.

### 4.1 Practical implications

The concentration of diabetes-related sickness absence and expenditures in large, industrialized, and highly populated voivodeships indicates that regional prevention, early diagnosis, and optimized disease management programmes could meaningfully reduce both absence rates and social insurance costs. Strengthening timely management of complications is essential to limit progression from short-term sick leave to longer-term rehabilitation benefits and permanent work incapacity. Integrating diabetes-related sickness absence indicators into national surveillance and workforce planning would help align health, labor, and social insurance policies and support more efficient allocation of resources to high-burden areas.

### 4.2 Limitations

Several limitations should be acknowledged. First, the study covers only persons participating in the social insurance system and therefore excludes self-employed individuals paying flat-rate contributions who are not entitled to sick leave benefits, farmers insured under the Agricultural Social Insurance Fund (KRUS), unemployed persons, and those working informally. This limits the generalizability of the findings to the insured employed population. Second, the analysis captures only sickness absence with diabetes recorded as the primary diagnosis causing work incapacity. Diabetes may also contribute to work disability through complications or as a comorbidity when another condition is recorded as the primary diagnosis; such cases were not captured, and the total diabetes-associated work disability burden may therefore be underestimated. Third, the study is descriptive, and causal inferences about the drivers of observed trends cannot be drawn from these aggregate registry data. Furthermore, the aggregate nationwide registry data did not include information on occupational sector, occupation, or job-specific characteristics, precluding assessment of the potential contribution of physical and mental workload, working hours, and other occupational factors to sickness absence patterns.

## 5 Conclusions

In Poland, diabetes-related sickness absence declined between 2020 and 2024, although the magnitude and pattern of this decline should be interpreted in the context of the COVID-19 pandemic and subsequent post-pandemic changes. The overall burden remained substantial, with rising social insurance expenditures, particularly for type 2 diabetes. Clear disparities by sex, age, and region were observed, including higher burden among men, increasing impact in young adults with type 1 diabetes, and growing long-term work incapacity related to type 2 diabetes. These findings highlight the need for targeted public health and workplace interventions addressing high-risk groups and the economic impact of diabetes on the labor force.

## Data Availability

The data analyzed in this study is subject to the following licenses/restrictions: Data are available from the corresponding author upon reasonable request. Requests to access these datasets should be directed to Kuba Sekowski kuba.sekowski@cmkp.edu.pl.
